# Robust Multi-Sensor Consensus Plant Disease Detection Using the Choquet Integral †

**DOI:** 10.3390/s23052382

**Published:** 2023-02-21

**Authors:** Cedric Marco-Detchart, Carlos Carrascosa, Vicente Julian, Jaime Rincon

**Affiliations:** 1 Valencian Research Institute for Artificial Intelligence, Universitat Politècnica de València, Camí de Vera s/n, 46022 Valencia, Spain; 2 Valencian Graduate School and Research Network of Artificial Intelligence, Universitat Politècnica de València, Camí de Vera s/n, 46022 Valencia, Spain

**Keywords:** smart agriculture, machine learning, EDGE-AI, sensors

## Abstract

Over the last few years, several studies have appeared that employ Artificial Intelligence (AI) techniques to improve sustainable development in the agricultural sector. Specifically, these intelligent techniques provide mechanisms and procedures to facilitate decision-making in the agri-food industry. One of the application areas has been the automatic detection of plant diseases. These techniques, mainly based on deep learning models, allow for analysing and classifying plants to determine possible diseases facilitating early detection and thus preventing the propagation of the disease. In this way, this paper proposes an Edge-AI device that incorporates the necessary hardware and software components for automatically detecting plant diseases from a set of images of a plant leaf. In this way, the main goal of this work is to design an autonomous device that allows the detection of possible diseases that can detect potential diseases in plants. This will be achieved by capturing multiple images of the leaves and implementing data fusion techniques to enhance the classification process and improve its robustness. Several tests have been carried out to determine that the use of this device significantly increases the robustness of the classification responses to possible plant diseases.

## 1. Introduction

The agri-food sector has always played a fundamental role in our society. It is essential because it supplies us with enough food to satisfy the nutritional needs of a constantly growing population. It is strategic because its activity has a significant impact in terms of economic production, employment generation, environmental management, and the maintenance of a living and balanced territory.

The availability of data on a farm is essential so that it can be analysed to generate information and knowledge to support producers in their decision-making and management of their operations. This includes using agricultural inputs more precisely, the ability to foresee the appearance of diseases, pests, or meteorological phenomena and thus adopt adaptive measures to reduce risks, the automation of tasks, and, therefore, a more efficient administration.

In this sense, techniques based on Artificial Intelligence (AI) have emerged as a valuable tool by providing mechanisms and procedures to facilitate the decision-making of specific tasks in the agri-food sector. Over the last few years, different approaches have tried to provide AI techniques for sustainable development in the farming sector, especially machine learning techniques. A deeper analysis and further reviews are presented in [1,2,3].

Another of the factors that most influence crop yields is the possible incidence of pests and diseases. To reduce their impact, farmers make regular use of phytosanitary products. In this way, the development of potentially dangerous populations can be controlled, thus ensuring production. This group also includes herbicides, which prevent competition for nutrients, water and the establishment of the main crops with other unwanted plants.

In many cases, given the uncertainty of when the pest will appear and how aggressive it will be, farmers often carry out preventive treatments. Over the last few years, the cost of these treatments has shown a clear upward trend; for example, in the EU, sales of active substances qualified as pesticides used in plant protection products exceed 350,000 t per year. Reducing the use of chemical pesticides by half by 2030 and the use of the most hazardous pesticides by 50% is one of the main objectives of the European Green Deal (https://ec.europa.eu/info/strategy/priorities-2019-2024/european-green-deal_en accessed on 5 February 2023).

Early detection of plant diseases through manual and visual inspections by experts or farmers has its limitations, including the dependency on a limited group of individuals and the potential for error due to the variety of plants and multiple diseases that can affect them. The automation of disease detection through the use of Artificial Intelligence techniques, specifically deep learning, offers numerous benefits [4]. Early treatment, as a result of early detection, reduces the need for chemical products and results in cost savings, preventing production losses, and contributing to environmental sustainability by avoiding the use of harmful phytosanitary products in the long term. The manual approach is also time-consuming and prone to human error, whereas AI automation offers a more efficient and reliable solution.

According to this, the use of Artificial Intelligence, particularly deep learning techniques, in plant disease detection has gained widespread popularity in recent years [5,6]. These approaches analyse and categorise plants to identify potential problems. Satellite and hyperspectral imaging is commonly utilised in agricultural analysis and plant disease detection. Satellite images provide a comprehensive view of the land and crop performance, whereas hyperspectral images offer a view beyond the visible spectrum, allowing for the use of tools such as the NDVI index to measure greenness and detect crop issues [7]. The main drawback of these approaches is the high cost of equipment (cameras and satellites) and processing of large images.

Another possible approach is to use closer images, such as leaves or sections of plants, to be analysed and classified to determine possible diseases [8,9]. Most of the analysed proposals in this line offer cloud services to perform detection. One of the problems we may encounter is the lack of connectivity in some rural regions and the need to transfer large amounts of data to perform the classification process in these services offered in the cloud. In this sense, the use of devices based on edge computing that detect diseases without the need for connections to cloud services and avoids continuous transfers of images over the network may be of greater interest.

Therefore, this paper presents an EDGE device that incorporates the necessary hardware and software components to automatically detect plant diseases from a set of images of the plant leaf. The device can be easily incorporated into an agricultural robot, a drone, or a tractor to facilitate automatic image acquisition in a crop field. Furthermore, the use of a set of images simultaneously, instead of just one, increases the robustness of the classifications, as demonstrated in the tests performed. This paper is an extended version of a previous paper published in the conference proceedings “Practical Applications of Agents and Multi-Agent Systems” [10] where a new device was developed and new designs and evaluations of the proposed solution were carried out.

The rest of the paper is structured as follows. Section 2 analyses previous related works, Section 3 presents the description of the proposed system, Section 4 describes the experiments carried out, and, finally, some conclusions are presented in Section 5.

## 2. Related Work

One of the main approaches to automatically detect plant diseases is through image analysis. This analysis may focus on different features such as geometry or colour. In some specific kinds of images, other indexes are also commonly used. So, for hyperspectral images, the NDVI index, which measures the level of green on images, is used. On the other hand, for visible range images, there are other alternative indexes such as the VARI index or the vNDVI index [11].

Identifying plant diseases automatically poses several challenges, as outlined by the review proposed in [12]. These challenges range from issues during the capture process, such as noise or fog over the camera, to the presence of unwanted information in the images, such as background, soil, or other plants. One way to deal with some of these problems is the pre-processing of images to not only eliminate spurious information, e.g., background segmentation or texture removal (smoothing [13,14]) or even image improvement (e.g., contrast enhancement [15]).

Apart from material-specific issues that may arise during image capture, another critical challenge is the potential existence of multiple diseases in a single plant. After image processing, automatic detection of plant diseases involves a classification task that can be approached using two main methods. The first involves classical Machine Learning (ML) techniques, where a set of features is extracted and chosen from the images, and then classified using techniques such as Support Vector Machines (SVM) [16,17], K-Means algorithm [18], or Random Forest [19], among others. These techniques need a very precise human-made solution (ground truth) and assistance to be performant. Furthermore, they must work well when there is a limited amount of data. Second, and currently used, one of the most popular approaches is the use of Deep Learning (DL) [20] and particularly Convolutional Neural Networks (CNN) [21,22] to train a model to identify the dataset classes. As is well known, even if there are increasingly more available images to work with, the quantity/quality is limited to learning from in a specific task. In those cases, Transfer Learning is used to build a network based on pre-trained information and adapted to the concerned task. These networks are pre-trained on large datasets, e.g., ImageNet [23]. This process takes the first layer of the trained network and removes the last layers adapting them to the specific task and training only these last steps. In this way, the specific task is not trained from scratch, and the computing time is shortened. The most common and efficient networks in the literature are *Alexnet* [24], *ResNet50* [25], *VGG16* [26], and *Inception V3* [27]. *EfficientNet* [28] can also be considered a group of networks, as there are eight types of subnets.

An alternative approach to mostly used network architecture is that of Capsule Networks [29] which fixes one of the significant drawbacks of standard CNN. CNNs do not consider the possible feature hierarchy in an image considering similar images as equal even when they are not. In the work presented by Samin et al. [30] the Capsule Network approach is used without using Transfer Learning, obtaining an accuracy of 93.07%.

More recently, a lightweight CNN approach based on Inception and Residual connection [31]. The proposed approach extracts better features from the input images. This is performed by replacing the standard convolution by a depth-wise separable convolution combined with a point-wise convolution, which results in fewer parameters as well as a speed-up in the processing. The resulting performance with the approach presented is 99.39% accuracy.

After studying the state-of-the-art, it can be seen that there is currently a multitude of proposals, most of them based on deep learning techniques that offer promising results from the existing datasets. However, there are specific gaps that we think should be analysed. On the one hand, some works suggest the need for image pre-processing before classification; in our opinion, this aspect should be studied in greater detail as it may allow for an improvement in the classification process. On the other hand, most of the works are evaluated against a so-called ideal dataset. Using more realistic datasets to validate existing models would allow for analysis of their possible robustness. Nevertheless, Ref. [32] is an interesting approach, but as they are working with infrared images, they would need to use infrared cameras when applied to the real world, which is expensive to deploy.

Apart from dealing with the above-mentioned gaps, our objective is to build a robust model capable of being deployed on an edge platform. In fact, our system, to be presented in the next section, focuses not only on the development of the software but also on the hardware infrastructure to give support to it.

## 3. System Description

**Figure 1:**
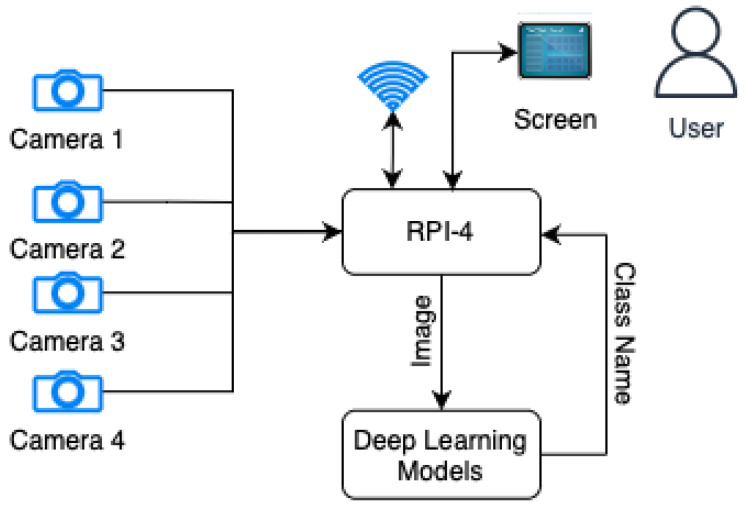
Description of the system.

In this section, the operation of the plant disease classification system using an EDGE device is explained in detail. The different software and hardware tools employed are also described. The proposed approach is shown in Figure 1.

**Figure 2:**
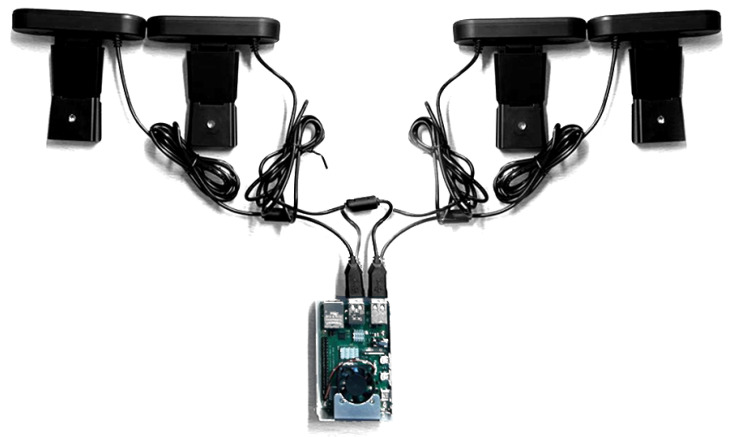
Hardware configuration.

The main components integrated into this prototype are the machine vision module, which is composed of four webcams (see Figure 2). We have the data processing module, which receives the four images from the cameras and reconstructs them to create an image composed of four images. The next element integrates the classification models to determine the plant’s disease. The system utilizes a WiFi communication system to send the classified data to the cloud, as well as a visualization system through an LCD screen. Further details regarding the hardware components and classification models are outlined in the subsequent sections. At a high level, the EDGE system employs cameras to capture four images from distinct angles, thus acquiring additional information and mitigating potential blind spots. The classification models are designed to identify whether the plant exhibits any of the 38 diseases present in the training database.

### 3.1. Hardware Description

This section describes the hardware used for plant disease recognition using a Raspberry 4. The Raspberry Pi 4 development system is a Broadcom BCM2711, Quad-core Cortex-A72 (ARM v8) 64-bit 1.5GHz SoC. We used an RPI-4 with 8GB SDRAM, IEEE 802.11ac wireless protocol, and Bluetooth 5.0, BLE for our experiments. It integrates four USB ports: two USB 3.0 and two USB 2.0.

**Figure 3:**
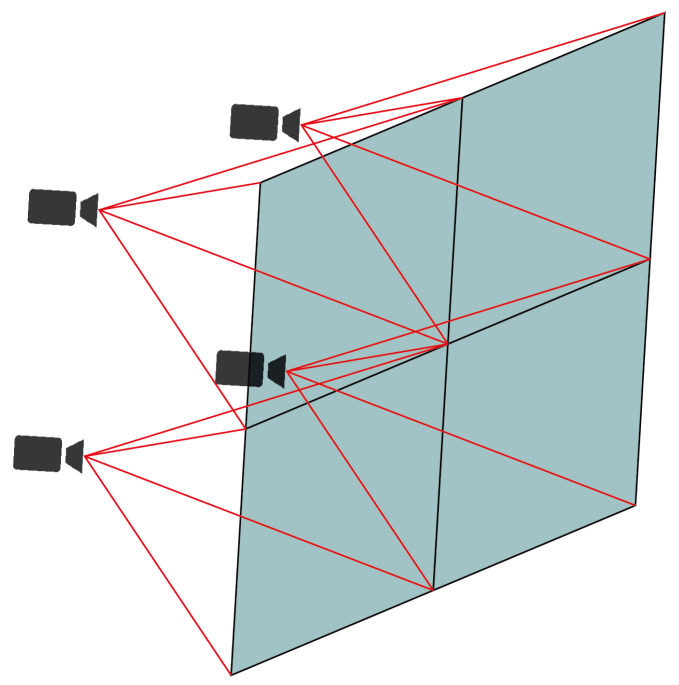
Array of cameras pointing at different views of the scene.

With this hardware configuration, it is possible to run trained TensorFlow lite models. To capture the images, we have used four 3-megapixel Logitech cameras with a resolution of 720p and a field of view (FOV) of 60 degrees. These cameras are spaced so that the FOVs of each bed overlap at a minimum (Figure 3).

Each of these images is resized to a size of 224 × 224. The models then analyse each of these images to determine what type of disease the plant has. The disease classification process will be described below.

The advantages of the proposed system are its low construction and maintenance costs. At the same time, it is a portable system of low weight, ideal for being integrated into either an unmanned aerial vehicle (UAV) or an unmanned ground vehicle (UGV). These can autonomously roam the field and report via GPS where the plants with the diseases are located. This would save time and money for the farmer; because they are completely autonomous, these systems can be programmed to start at any time of the day.

### 3.2. Software Description

In the following section, we will describe the different software tools used. The system proposed in this work uses deep learning techniques using a MobileNet v2 network for plant disease classification. This system is embedded in a Raspberry Pi 4, which integrates the trained ML model.

The MobileNet v2 [33] architecture, one of the most widely used neural networks in mobile applications, is based on an inverted residual structure in which the input and output of the residual block are thin bottleneck layers. Unlike other models with these features, MobileNet v2 employs lightweight convolutions in depth to filter features in the intermediate expansion layer. Additionally, to preserve its representational power, the network has eliminated nonlinearities in the narrow layers. Research presented by Kristiani et al. [34] showed from the experiments that Mobilenet outperforms Inception in terms of speed, accuracy and file size. The speed in Inception V3 is 9 frames per second, while that value in Mobilenet is 24 frames per second.

The structure of the compiled model is depicted in Table 1, and it remains consistent across all the configurations (Table 2) utilised in this study. Once the training process is complete, the model is stored as a *.tflite file and embedded in the Raspberry Pi 4. In the event of a classification improvement or the addition of a new disease class, this model can be effortlessly updated.

The four cameras of the prototype capture four different images of the plant to be analysed. These four images are preprocessed to make them compatible with the trained model. This preprocessing consists of resizing the images from 1280 × 720 to 224 × 224. Each newly resized image is used as input for the classification model. In this way, the system can analyse the plant from four different points of view. These four images are used by the model one at a time. This result in 38 probabilities that are added to a 38 × 4 matrix (38 classes and four cameras). This matrix is then used as input for the data fusion algorithm to obtain the final probabilities, which is explained in the next section. We used two datasets to train and validate the model: the PlantVillage and the extended PlantVillage. The PlantVillage contains leaf images with a homogeneous background. However, the extended PlantVillage is formed by the original images but with a synthetic background that simulates a field to add noise to the image and make it more realistic. This test aims to check the model’s robustness and to see if the model performs well against this more realistic dataset.

## 4. Experimental Setup

In this Section, we analyse the performance of the different configurations used. First, in Section 4.1, we present the data set used for our experiments and the measures used to quantify the results obtained. Second, in Section 4.2, we present the quantitative results of our experiments.

In the first round of experiments, we test two well-known mobile-oriented device networks. Then we select the best performer network and tested it with our consensus classification approach. The experiment employed the Mobilenet V2 and NasnetMobile networks, and several hyperparameters were established to train both networks, while some hyperparameters remained constant across all experiments, others were altered to determine the optimal settings for the best results. To avoid overfitting, a maximum of seven epochs was selected. The learning rate for all models remained identical, and data augmentation and fine-tuning were activated or deactivated, depending on the specific experiment. Table 2 illustrates the hyperparameter configurations.

To be able to treat the four-grid system, we propose using an aggregation procedure to summarise the data captured by the setup. Every set of images captured by the cameras is considered a batch where the corresponding trained network is applied to each one. Then the output of each image is aggregated to produce one result that better represents all of the initial data. To proceed with the data fusion, we need some definitions:

**Definition** **1**  ([35])**.** *A mapping $M:{\left[0,1\right]}^{n}\to \left[0,1\right]$ is an aggregation function if it is monotone non-decreasing in each of its components and satisfies the boundary conditions, M(0)=M(0,0,…,0)=0 and M(1)=M(1,1,…,1)=1.*

**Definition** **2.** *A function $m:2^{N}\to \left[0,1\right]$ is a fuzzy measure if, for all X,Y⊆N, it satisfies the following properties:*

1. *Increasingness: if X⊆Y, then m(X)≤m(Y);*
2. *Boundary conditions: m(∅)=0 and m(N)=1.*

An example of a commonly used fuzzy measure is the power measure, which we use in this work:

(1) $$m_{q}\left(X\right)={\left(\frac{\left|X\right|}{n}\right)}^{q},\;with\;q>0,$$

where |X| is the number of elements to be aggregated, *n* the total number of elements and q>0. We have selected this measure due to the performance obtained in terms of accuracy in classification problems [36,37].

**Definition** **3**  ([35])**.** *Let $m:2^{N}\to \left[0,1\right]$ be a fuzzy measure. The discrete Choquet integral of $x=\left(x_{1},\cdots ,x_{n}\right)\in {\left[0,1\right]}^{n}$ with respect to m is defined as the function $C_{m}:{\left[0,1\right]}^{n}\to \left[0,1\right]$, given by*

$$C_{m}\left(x\right)=\sum_{i=1}^{n}\left(x_{\left(i\right)}-x_{\left(i-1\right)}\right)\cdot m\left(A_{\left(i\right)}\right),$$

*where $\left(x_{\left(1\right)},\ldots ,x_{\left(n\right)}\right)$ is an increasing permutation on the input x, that is, $x_{\left(1\right)}\leq \ldots \leq x_{\left(n\right)}$, with the convention that $x_{\left(0\right)}=0$, and $A_{\left(i\right)}=\left\{\left(i\right),\cdots ,\left(n\right)\right\}$ is the subset of indices of the n−i+1 largest components of x.*

The Choquet integral is idempotent and presents an averaging behaviour. Observe that the Choquet integral is defined using a fuzzy measure, permitting to consider the relation between the elements considered to be aggregated (i.e., the components of an input x).

In our experiments, after applying each trained model to the images, the output shows the membership of each image to the 38 classes of the dataset. Then, we aggregate ordering increasingly for each class probability output and apply the Choquet integral to obtain the final membership probability for each class. Finally, we take the maximum response to select the class of the image batch.

### 4.1. Dataset and Quantification of the Results

In this work, we use two datasets, one to train and validate our model and a derived one where some modifications are added to the first one to simulate the real-environment process of the proposed approach. This study is in an early stage, so all the processes have been performed in a controlled environment with already captured images. In a future second stage, the objective is to test our proposed system in a real environment.

**Figure 4:**
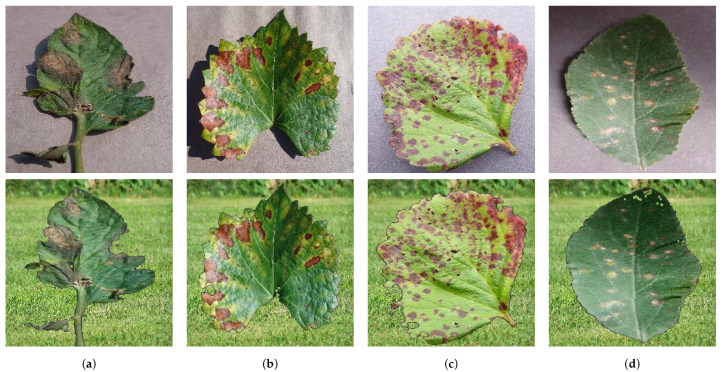
Example of images included in the PlantVillage dataset [38] from different plant diseases along with their equivalent in the Synthetic PlantVillage dataset. (**a**) Tomato—blight; (**b**) grape—esca; (**c**) strawberry—scorch; (**d**) apple—rust.

The primary dataset used in this study is known as PlantVillage (PV) [38], comprising roughly 87,000 RGB images of healthy and diseased crop leaves, which are categorized into 38 distinct classes. The images are captured from individual leaves of each plant and disease against a consistent background. To facilitate training, the dataset was split into three subsets, with an 80/20 ratio: 80% for training, 10% for testing, and the remaining 10% for validation. Raw images (Figure 4) were utilized for model training and validation without undergoing any image pre-processing.

The second dataset is called SyntheticPlantVillage (SynPV). It is a modified version where the background of the images has been removed and filled with a grass background simulating a real scenario for the leaves. This second dataset is used to put our proposal to the test analysing its robustness in a real-like scenario. We simulate the camera grid capture taking batches of the same class images and aggregate its classification to obtain a consensus decision about the final disease class.

To interpret the results obtained in the confusion matrix, we use the following Precision/Recall measures:

$$Prec=\frac{TP}{TP+FP},\;Rec=\frac{TP}{TP+FN},\;F_{\beta }=\left(1+\beta^{2}\right)\frac{Prec\cdot Rec}{\beta^{2}\cdot Prec+Rec}.$$

We select the values of β=0.5 and β=1 as it is the most commonly used in the literature.

### 4.2. Experimental Results

In this section, we present the outcomes obtained with various configurations listed in Table 2. Quantitative results are shown in Table 3. Furthermore, we carried out real-world simulations in a controlled environment of our models using a Raspberry Pi 4 and a four-camera setup, as outlined in the proposed solution.

**Figure 5:**
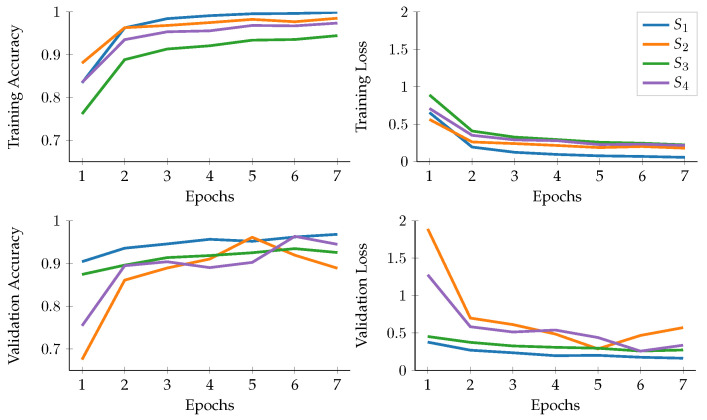
Training and validation accuracy and loss obtained with the different configurations from Table 2.

The behaviour of each configuration used can be seen in Figure 5, which depicts how they perform similarly across various epochs. During the validation phase, on the one hand, $S_{2}$ and $S_{4}$ exhibit an unpredictable pattern, starting with low accuracy, then suddenly increasing, fluctuating until the end of the training. On the other hand, the performance of $S_{1}$ and $S_{3}$ is more consistent, reaching stability by the end of the epochs. These trends are further reflected in the quantitative results obtained from the testing phase.

As shown in Table 3, the best results (validated on the training machine) are achieved with configuration $S_{1}$, without the use of transfer learning or data augmentation. The second-best outcome is produced by $S_{3}$, which only employs data augmentation. This observation suggests that the use of MobileNet v2 with transfer learning for the specific task of identifying plant disease does not enhance performance and, in fact, results in a decrease in all measures. The use of transfer learning has a negative impact on the results, as the weights learned from the ImageNet dataset, which has a large number of classes, affect the outcomes in $S_{2}$ and $S_{4}$.

As we can observe, using our new proposal, with a four-grid camera to capture plant images, increases the system’s scores. In general, all the configurations benefit from the new setup, and in the case of $S_{1}$, which remains the best performer, the result of $F_{1}$ obtains a 98.2% performance. To prove the presented approach’s benefits, we tested it over a SynPv dataset, measuring the approach’s robustness. This second experiment shows that comparing the results with SynPV using the initial approach with one camera, the best performer, $S_{1}$, decays to 64.4% but using the new setup increases considerably almost to an 80% performance in terms of $F_{1}$. These results indicate that our new approach increases the robustness of the system, even when artefacts and unwanted information are present in the image.

The model training and validation were performed on a machine equipped with an Intel i5-9500 processor running at 4.4 GHz, 16 GB of RAM, and operating on Ubuntu 20.04.4 LTS. For the actual validation of the model, a Broadcomm BCM2835 ARM11 1 GHz with a VideoCore IV GPU and 512 MB of RAM was utilised.

Table 4 presents the top-performing outcomes of the experiments conducted using the NasNetMobile neural network, which was validated with the plant village dataset (PV) and Synthetic PlantVillage (SynPV). Like the MobileNet, the NasNetMobile [39] is a convolutional neural network (CNN) model created to carry out image classification tasks on mobile devices. The NasNetMobile’s architecture relies on a search algorithm that utilizes reinforcement learning techniques to identify the optimal network structure for a given task, thereby eliminating the need for manual tuning by developers. Similar to the MobileNet V2, transfer learning with the NasNetMobile network for identifying plant diseases does not enhance its performance; instead, it leads to a decrease in results. The use of transfer learning has an impact on the classification process, which may be attributed to the training of the ImageNet dataset and the types of images utilized to obtain the weights. As a result, the classification values are influenced in the experiments that leverage transfer learning (S2 and S8).

**Figure 6:**
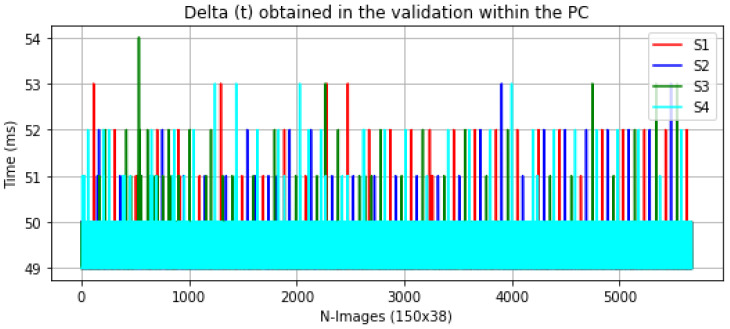
Delta (t) obtained in the validation within PC.

**Figure 7:**
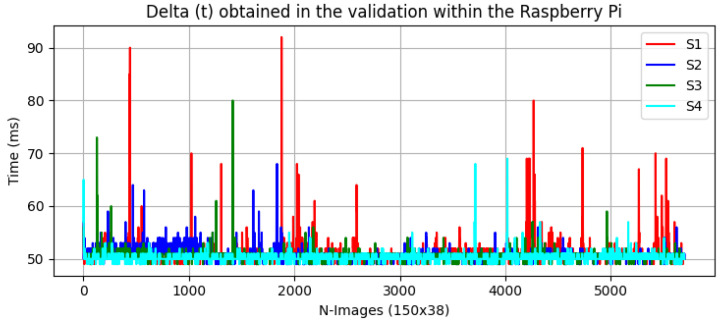
Delta (t) obtained in the validation within the Raspberry Pi.

Figure 6 and Figure 7 below show the Δ(t) of the validation process between the computer and the Raspberry Pi for each experiment.

As expected, the validation times on the computer were more consistent owing to its processor and memory characteristics. However, the validation times on the Raspberry Pi were more erratic. Experiment $S_{1}$ had the highest peak time, with a maximum runtime of roughly 90 ms. This could be because this experiment was trained from scratch, with no data augmentation or fine-tuning, indicating that this model does not employ pre-trained weights like the other models. Additionally, its weight in bytes might be larger than the other models, resulting in higher memory storage and resource utilization. Despite these variances, the models are still appropriate for our application.

## 5. Conclusions

This study introduces a low-cost smart device that allows for the detection of possible diseases, increasing the robustness of the classification process by capturing several images of the leaves and integrating techniques based on data fusion. The device has been developed using a Raspberry Pi 4 and a four-camera array and incorporates a deep learning model to classify and display information on an LCD screen and is easily mountable on drones, robots, or agricultural machinery. The performance measures and tests have been performed in a controlled environment with modified images to simulate outdoor spaces and evaluate the system’s robustness.

The device offers a more efficient method of visualizing plant disease spots, reducing costs by eliminating the excessive use of fungicides, pesticides, and herbicides.

The results show how the proposed device demonstrates that current EDGE technology permits carrying out plant disease classification and detection systems, considerably lowering the usage cost as no images are transmitted over an Internet connection. Moreover, it allows farmers to perform a pre-analysis of possible diseases that may be present in their plants.

We also show that our system is more robust than a single-camera setup, obtaining better results than the original and synthetic datasets, where noise and unwanted intimation have been added to the images.

Future work aims to put the proposed system to the test in a natural environment mounted on a robot or drone. Further, another experimental path is to detect changes in plant disease severity over time by adapting models to identify, classify, and assess the extent of disease progression following the disease evolution cycle. Furthermore, determining the effects of multiple infections on plants is also of interest.

## Figures and Tables

**Table 1:** Main characteristics of the employed model.

Layer (Type)	Param	Output Shape#
keras_layer (KerasLayer)	2,257,984	(None, 1280)
flatten (Flatten)	0	(None, 1280)
dense (Dense)	655,872	(None, 512)
dropout (Dropout)	0	(None, 512)
dense_1 (Dense)	19,494	(None, 38)

**Table 2:** Hyperparameters used to configure the neural net used in the experiments.

#	Net Type	N-Epochs	Learning Rate	Transfer Learning	Data Augmentation	Data Set
$S_{1}$	Mobilenet V2	7	0.001	✗	✗	Raw Image
$S_{2}$	Mobilenet V2	7	0.001	✔	✗	Raw Image
$S_{3}$	Mobilenet V2	7	0.001	✗	✔	Raw Image
$S_{4}$	Mobilenet V2	7	0.001	✔	✔	Raw Image
$S_{5}$	NasNetMobile	7	0.001	✗	✗	Raw Image
$S_{6}$	NasNetMobile	7	0.001	✔	✗	Raw Image
$S_{7}$	NasNetMobile	7	0.001	✗	✔	Raw Image
$S_{8}$	NasNetMobile	7	0.001	✔	✔	Raw Image

**Table 3:** Resulting test performance of the model trained with the parameters in Table 2 over the original dataset PlantVillage (PV) and the synthetic one (SynPV), simulating grass on the background. Results are shown with the original proposal (Prec, Rec, $F_{0.5}$, and $F_{1}$) and the new one using the array of cameras ($Prec^{\prime }$, $Rec^{\prime }$, $F_{0.5}^{\prime }$, and $F_{1}^{\prime }$).

#	Dataset	Prec	${Prec}^{\prime }$	Rec	${Rec}^{\prime }$	$F_{0.5}$	$F_{0.5}^{\prime }$	$F_{1}$	$F_{1}^{\prime }$
$S_{1}$	PV	**0.881**	0.986	**0.904**	0.980	**0.882**	0.985	**0.886**	0.982
	SynPV	**0.744**	0.872	**0.665**	0.819	**0.669**	0.811	**0.644**	0.796
$S_{2}$	PV	0.829	0.927	0.852	0.945	0.813	0.923	0.807	0.923
	SynPV	0.616	0.676	0.465	0.553	0.456	0.546	0.423	0.510
$S_{3}$	PV	**0.881**	0.986	0.897	0.978	0.881	0.983	0.883	0.980
	SynPV	0.719	0.840	0.650	0.790	0.643	0.769	0.623	0.758
$S_{4}$	PV	0.852	0.947	0.862	0.940	0.836	0.932	0.831	0.927
	SynPV	0.658	0.487	0.375	0.383	0.419	0.368	0.358	0.337

**Table 4:** Resulting test performance of the model trained with the parameters in Table 2 over the original dataset PlantVillage (PV) and the synthetic one (SynPV), simulating grass on the background. Results are shown with the original proposal using a single camera.

#	Dataset	Prec	Rec	$F_{0.5}$	$F_{1}$
$S_{5}$	PV	0.813	0.851	0.812	0.816
	SynPV	0.630	0.553	0.558	0.531
$S_{6}$	PV	0.711	0.843	0.844	0.840
	SynPV	0.632	0.343	0.368	0.370
$S_{7}$	PV	0.787	0.810	0.780	0.779
	SynPV	0.632	0.535	0.527	0.504
$S_{8}$	PV	0.716	0.827	0.813	0.914
	SynPV	0.672	0.490	0.492	0.499
